# Association between body composition in early pregnancy and the risk of gestational diabetes mellitus

**DOI:** 10.3389/fnut.2025.1565986

**Published:** 2025-05-21

**Authors:** Yan Li, Bin Zhao, Yang Tian, Yang Li, Xia Li, Hongmei Guo, Li Xiong, Jiaying Yuan

**Affiliations:** ^1^Department of Clinical Nutrition, Chengdu Shuangliu District Maternal and Child Health Care Hospital, Chengdu, China; ^2^Department of Obstetrics, Chengdu Shuangliu District Maternal and Child Health Care Hospital, Chengdu, China; ^3^Department of Clinical Pharmacy, Chengdu Shuangliu District Maternal and Child Health Care Hospital, Chengdu, China; ^4^Department of Science and Education, Chengdu Shuangliu District Maternal and Child Health Care Hospital, Chengdu, China

**Keywords:** gestational diabetes mellitus, body composition, body fat percentage, fat mass, fat free mass

## Abstract

**Background:**

Gestational diabetes mellitus (GDM) is a common complication during pregnancy that poses serious health risks to both mothers and their offspring. Risk factors for GDM, such as obesity, have been extensively reported. However, the association between body composition and GDM risk remains unclear. Therefore, we conducted a retrospective cohort study to investigate the relationship between body composition in early pregnancy and the risk of developing GDM.

**Methods:**

A total of 3,159 pregnant women were enrolled between June 2020 and December 2021, with 280 (10.43%) diagnosed with GDM. Bioelectrical impedance analysis (BIA) was used to measure the percentage of body fat (PBF), fat mass (FM), fat-free mass (FFM), and lean mass (LM). Logistic regression and restricted cubic spline (RCS) analyses were performed to examine the associations between body composition and GDM risk.

**Results:**

Compared with the bottom tertile, the top tertile levels of PBF and FM were significantly associated with an increased risk of GDM, with adjusted odds ratios (ORs) and corresponding 95% confidence intervals (95% CI) of 1.77 (1.13, 2.77) and 1.99 (1.23, 3.20), respectively. Each standard deviation (SD) increase in PBF and FM was associated with a 31% (95% CI: 1.07–1.60) and 27% (95% CI: 1.03–1.57) increased risk of GDM, respectively. RCS analysis indicated that the risk of GDM continuously increased with higher levels of PBF and FM, whereas it decreased with FFM and LM (*p*-overall < 0.001, *p*-non-linear range: 0.073–0.924). These findings provide important threshold values in predicting GDM risk, specifically 24.74% for PBF, 13.13 kg for FM, 39.81 kg for FFM, and 36.74 kg for LM.

**Conclusion:**

The risk of GDM is positively associated with PBF and FM whereas negatively associated with FFM and LM.

## Introduction

Gestational diabetes mellitus (GDM) is a common complication characterized by impaired glucose metabolism during pregnancy, and it can adversely affect the health of both mothers and their offspring (1). GDM elevates the risk of adverse pregnancy outcomes, including fetal dysplasia, neonatal hypoglycemia, and preterm birth (2, 3), and is also associated with an elevated risk of long-term insulin resistance and type 2 diabetes (4, 5). However, the etiology of GDM is complex and remains incompletely understood. Further research is urgently needed to identify the potential risk factors for GDM.

Pre-pregnancy obesity has been demonstrated to play a significant role in the development of GDM through multiple pathways (1). Body mass index (BMI) is commonly used to identify women at risk of developing GDM during pregnancy; however, it cannot provide accurately distinguish between fat mass and lean mass (6). Studies have shown that both adipose and muscle tissues contribute to insulin sensitivity (7). During pregnancy, maternal metabolism accelerates to support the rapid growth and development of the fetus, and maternal intestinal fat absorption capacity increases, resulting in a greater accumulation of visceral and subcutaneous fat compared to pre-pregnancy levels (8). A cohort study involving 627 women indicated that visceral adipose tissue in early pregnancy was a better predictor of GDM risk than the traditionally used BMI (9).

Body composition, consisting of muscle, fat, and bone mass, can be easily measured using bioelectric impedance analysis (BIA). Excessive fat accumulation may induce chronic inflammation in adipose tissue, dysfunction of pancreatic β-cells, and ultimately, systemic insulin resistance (10). Evidence has shown that individuals with normal weight but a high body fat percentage have a greater risk of cardiovascular disease and metabolic syndrome compared to those with both normal weight and normal body fat percentage (11, 12). A 15-year follow-up cohort study conducted in the Japanese population found that leg fat percentage was negatively associated with diabetes risk in both men and women (13). Additionally, pregnancy complications such as preeclampsia (14) and gestational hypertension (15) are also associated with body composition. However, only a few studies have explored the association between body composition and GDM risk (6). The role of body composition in early pregnancy in predicting GDM during the second trimester remains unclear.

Body composition distribution also changes with age, characterized by an increase in total fat content (especially abdominal fat), along with a decrease in lean mass and bone density (16). Additionally, certain medical conditions (such as hypothyroidism and polycystic ovary syndrome) may contribute to increased fat accumulation (17, 18). Unhealthy body composition can be effectively improved through lifestyle management. Evidence suggests that interventions such as reducing energy intake and increasing physical activity levels can positively influence body composition (19). Early identification of associations between abnormal body composition and GDM can facilitate its prevention. Therefore, the aim of our study was to explore the relationship between body composition in early pregnancy and the risk of developing GDM.

## Methods

### Study design

This retrospective cohort study recruited women in early pregnancy (6–13 weeks of gestation) who registered and delivered at Chengdu Shuangliu District Maternal and Child Health Care Hospital (Sichuan Province, China) between June 2020 and December 2021. Written informed consent was obtained from all participants. The study was approved by the Ethics Committee of Chengdu Shuangliu District Maternal and Child Health Care Hospital (Approval No. ky202404) and conducted in accordance with the Declaration of Helsinki of the World Medical Association.

### Participants

A total of 3,249 eligible pregnant women aged 18–45 years at 6–13 weeks of gestation were initially recruited. Among these participants, 90 pregnant women were excluded due to the following conditions: (1) chronic metabolic diseases diagnosed before pregnancy, including previous GDM or other types of diabetes (*n* = 4), polycystic ovary syndrome (PCOS) (*n* = 17), thyroid dysfunction (*n* = 16), chronic nephritis (*n* = 5), and heart disease (*n* = 1); (2) infectious diseases (*n* = 33), such as acquired immune deficiency syndrome (AIDS) (*n* = 2), syphilis (*n* = 5), hepatitis (*n* = 22), severe pneumonia (*n* = 2), pulmonary tuberculosis (*n* = 1), and myocarditis (*n* = 1); and (3) incomplete body composition information (*n* = 14). Ultimately, 3,159 women were included in the study. All participants underwent an oral glucose tolerance test between 24 and 28 weeks of gestation.

### Collection of basic information

The collection of basic information and measurement of physical parameters were performed by trained medical professionals, including doctors and nurses. The demographic characteristics and medical histories of pregnant women were obtained through face-to-face interviews during the first prenatal visit. Collected data included maternal age, education level (junior, senior, college), ethnicity (Han, other), household registration type (rural, urban), gravidity (<3, ≥3), parity (primiparity, multiparity), last menstrual period, and a previous disease history. Height and weight were measured using an electronic scale with an accuracy of 0.1 cm and 0.1 kg, respectively. BMI was calculated as early pregnancy weight (kg) divided by height squared (m^2^) and classified into three categories: underweight (<18.5 kg/m^2^), normal (18.5–23.9 kg/m^2^), and overweight/obesity (≥24 kg/m^2^). Maternal age was categorized into two groups: <35 years and ≥35 years.

### Collection of body composition data

Body composition in early pregnancy was assessed using BIA (NAQ-P, Si Hai Hua Chen, Inc., China). Before measurement, participants were instructed to wear light clothing without any metal accessories and stand barefoot on the metal plates of the instrument. They were then asked to lightly hold the metal electrodes with both hands. Data were recorded once the measurement was completed. Body fat mass (FM), fat-free mass (FFM), lean mass (LM), percentage of body fat (PBF), and basal metabolic rate (BMR) were recorded. PBF was calculated as the ratio of FM to total weight multiplied by 100%.

### Diagnosis of GDM

The diagnosis of GDM followed the guidelines established by the International Association of Diabetes and Pregnancy Study Groups (20). An oral glucose tolerance test was performed at 24–28 weeks of gestation, for which the participants were instructed to fast overnight for 8–12 h and then consume 300 mL of glucose solution containing 75 g glucose within 5 min before 9:00 AM. Blood samples were collected at fasting and 1 h and 2 h after glucose loading. GDM was diagnosed if any of the fasting, 1-h, or 2-h post-load blood glucose values reached or exceeded 5.1, 10.0, or 8.5 mmol/L, respectively.

### Statistical analysis

Data processing and analysis were performed using RStudio software, version 4.3.0 (RStudio, Inc., United States).

Continuous variables with normal distribution are expressed as means with standard errors (SE), while categorical variables are presented as frequencies with percentages (%). Non-normally distributed data are displayed as medians (interquartile ranges, IQR). Between-group differences in continuous and categorical variables were assessed using the independent samples t-test and chi-square test, respectively. Given the skewed distribution of the data, Spearman’s correlation coefficients were calculated to assess correlations between early-pregnancy body composition and blood glucose values at different time points. Additionally, the continuous body composition variables (PBF, FM, FFM, and LM) were divided into tertiles, with the first tertile serving as the reference group. Binary logistic regression analysis was subsequently conducted to estimate odds ratios (ORs) and corresponding 95% confidence intervals (95% CIs) for the risk of GDM associated with these body composition variables in early pregnancy. Restricted cubic spline (RCS) with three knots was conducted to assess dose–response relationships between body composition parameters and GDM risk. A two-tailed *p*-value < 0.05 was considered statistically significant. Model 1 represented unadjusted univariate analysis, whereas Model 2 was adjusted for potential confounding variables, including maternal age, education level, ethnicity, household registration type, early-pregnancy BMI, gestational weight gain, gravidity, and parity.

## Results

This study included 3,159 pregnant women with a mean age of 27.77 ± 3.99 years, among whom 280 (10.43%) were diagnosed with GDM (Table 1). Pregnant women with GDM had significantly higher maternal age (29.58 ± 4.43 vs. 27.59 ± 3.90 years, *p* < 0.001), early-pregnancy BMI (22.72 ± 2.91 vs. 21.72 ± 2.76 kg/m^2^, *p* < 0.001), and gravidity (≥3 pregnancies: 44.34% vs. 32.78%, *p* < 0.001) compared with normal pregnant women. However, the gestational weight gain in the GDM group was significantly lower than that in the normal group (5.01 ± 3.11 vs. 7.04 ± 3.79 kg, *p* < 0.001). No significant differences were observed in the baseline gestational weeks, ethnicity, household registration type, education level, or parity between the GDM and normal groups (all *p* > 0.05).

**Table 1:** Basic information among 3,159 participants according to GDM status.

Variables	Overall (*N* = 3,159)	None-GDM (n1 = 2,879)	GDM (n2 = 280)	*p*-values
Gestational weeks	10.43 (1.91)	10.43 (1.93)	10.48 (1.73)	0.684
Maternal age, years	27.77 (3.99)	27.59 (3.90)	29.58 (4.43)	**<0.001**
Maternal age, *n* (%)				**<0.001**
≤35	3,049 (96.52)	2,796 (97.12)	253 (90.36)	
>35	110 (3.48)	83 (2.88)	27 (9.64)	
Nation (Han), *n* (%)	3,104 (98.26)	2,828 (98.23)	276 (98.57)	0.858
Education level, *n* (%)				
Junior	639 (20.35)	559 (19.54)	80 (28.67)	
Senior	2014 (64.14)	1868 (65.29)	146 (52.33)	**<0.001**
College	487 (15.51)	434 (15.17)	53 (19.00)	
Account (Rural), *n* (%)	2,141 (67.77)	1935 (67.21)	206 (73.57)	**0.035**
Gravidity, *n* (%)				
<3	2,393 (66.14)	2,207 (67.22)	186 (45.66)	**0.001**
≥3	766 (33.86)	672 (32.78)	94 (44.34)	
Parity, *n* (%)				
Primiparity	2,192 (57.25)	2012 (57.71)	180 (52.83)	0.196
Multiparity	967 (42.75)	867 (42.29)	100 (47.17)	
Early pregnancy BMI, kg/m2	21.81 (2.79)	21.72 (2.76)	22.72 (2.91)	**<0.001**
Early pregnancy BMI, kg/m^2^				**<0.001**
<18.5	250 (7.91)	234 (8.13)	16 (5.71)	
18.5 ~ 23.9	2,261 (71.57)	2079 (72.21)	182 (65.00)	
≥24	648 (20.51)	566 (19.66)	82 (29.29)	
Gestational weight gain, kg	6.78 (3.78)	7.04 (3.79)	5.01 (3.11)	**<0.001**
FBG, mmol/L	4.03 (0.38)	3.99 (0.32)	4.48 (0.59)	**<0.001**
OGTT-1 h, mmol/L	7.23 (1.67)	6.94 (1.39)	10.19 (1.37)	**<0.001**
OGTT-2 h, mmol/L	6.29 (1.37)	6.04 (1.08)	8.90 (1.35)	**<0.001**

Data are presented as mean ± standard deviation (SD) for continuous variables and frequency (%) for categorical variables.

GDM, gestational diabetes mellitus; N/n, numbers of subjects; BMI, body mass index; OGTT, oral glucose tolerance test; SD, standard deviation. Bold values represent statistical significance.

Table 2 shows the comparison of early pregnancy body composition parameters between the GDM and normal groups. Pregnant women with GDM had significantly higher levels of PBF, FM, FFM, and LM compared with normal pregnant women (all *p <* 0.005).

**Table 2:** Comparison of body composition between GDM group and None-GDM group.

Variables	Overall (*N* = 3,159)	None-GDM (n1 = 2,879)	GDM (n2 = 280)	*P*-values
PBF, %	25.16 (4.49)	25.03 (4.45)	26.47 (4.72)	**<0.001**
FM, kg	13.87 (4.18)	13.76 (4.14)	15.03 (4.41)	**<0.001**
FFM, kg	40.24 (3.94)	40.19 (3.94)	40.74 (3.85)	**0.026**
LM, kg	36.94 (4.46)	36.88 (4.48)	37.51 (4.30)	**0.024**

Data are presented as mean (SD).

N/n, numbers of subjects; GDM, gestational diabetes mellitus; n, numbers of subjects; PBF: percentage of body fat; FM, body fat mass; FFM, fat free mass; LM, lean mass. Bold values represent statistical significance.

Normality tests indicated that blood glucose values and body composition indicators were skewed in distribution (Supplementary Table 1 and Supplementary Figure 1). Therefore, Spearman correlation analyses were conducted. As shown in Table 3, Spearman’s correlation analysis revealed positive correlations between early pregnancy PBF, FM, FFM, and LM levels and blood glucose values measured during the oral glucose tolerance test at 24–28 weeks of pregnancy (*r*: 0.07–0.18, all *p* < 0.001).

**Table 3:** Correlation between body composition index and glucose values in OGTT.

Body composition markers	OGTT-fasting		OGTT-1 h		OGTT-2 h	
	β (95% CI)	*P*-values	β (95% CI)	*P*-values	β (95% CI)	*P*-values
PBF, %	0.18 (0.16, 0.23)	**<0.001**	0.13 (0.11, 0.18)	**<0.001**	0.15 (0.11, 0.18)	**<0.001**
FM, kg	0.18 (0.16, 0.23)	**<0.001**	0.14 (0.11, 0.18)	**<0.001**	0.15 (0.11, 0.18)	**<0.001**
FFM, kg	0.10 (0.09, 0.15)	**<0.001**	0.09 (0.06, 0.13)	**<0.001**	0.07 (0.04, 0.10)	**<0.001**
LM, kg	0.11 (0.09, 0.16)	**<0.001**	0.10 (0.07, 014)	**<0.001**	0.08 (0.05, 0.12)	**<0.001**

Data are presented as β with 95% confidence intervals.

OGTT, oral glucose tolerance test; PBF, percentage of body fat; FM, body fat mass; FFM, fat free mass; LM, lean mass. Bold values represent statistical significance.

Generally, a tolerance value of <0.2 or a variance inflation factor (VIF) of >5 indicates multicollinearity among independent variables. Based on these criteria, the collinearity between BMI and body composition parameters (PBF, FM, FFM, LM) was considered acceptable (Supplementary Table 2). Table 4 presents the ORs and corresponding 95% CIs for GDM risk according to early-pregnancy levels of PBF, FM, FFM, and LM. When modeling one body composition measurement (e.g., PBF), other measurements were not included simultaneously. Compared with the lowest tertile, the multivariable-adjusted ORs (95% CIs) for GDM in the highest tertile were 1.77 (1.13, 2.77) for PBF and 1.99 (1.23, 3.20) for FM (both *p* for trend < 0.001). Additionally, each one-standard deviation increase in early-pregnancy PBF and FM was associated with a 31% (OR = 1.31, 95% CI: 1.07–1.60) and 27% (OR = 1.27, 95% CI: 1.03–1.57) higher risk of GDM, respectively.

**Table 4:** Odds ratios and corresponding 95% confidence intervals for GDM according to tertiles of body composition in early pregnancy.

Variable	ORs (95% CIs) for GDM			Per 1 SD increase	*P* for Trend
	Tertile 1	Tertile 2	Tertile 3		
PBF, %	<22.60	22.60–27.20	≥27.30		
Case/total (%)	68/1036 (6.56)	89/1172 (7.59)	123/951 (12.93)		–
Model 1	Reference	1.17 (0.84, 1.63)	**2.11 (1.56, 2.90)**	**1.37 (1.21, 1.54)**	**<0.001**
Model 2	Reference	0.90 (0.6, 1.37)	**1.77 (1.13, 2.77)**	**1.31 (1.07, 1.60)**	**<0.001**
FM, kg	<11.50	11.50–15.30	≥15.40		
Case/total (%)	66/1017 (6.49)	97/1205 (8.05)	117/937 (12.49)		–
Model 1	Reference	1.26 (0.91, 1.75)	**2.06 (1.50, 2.83)**	**1.33 (1.18, 1.49)**	**<0.001**
Model 2	Reference	1.08 (0.72, 1.66)	**1.99 (1.23, 3.20)**	**1.27 (1.03, 1.57)**	**<0.001**
FFM, kg	<38.50	38.50–41.50	≥41.60		
Case/total (%)	75/1049 (7.15)	112/1138 (9.84)	93/972 (9.57)		–
Model 1	Reference	1.42 (1.05, 1.93)	**1.37 (1.00, 1.89)**	**1.14 (1.01, 1.29)**	**<0.001**
Model 2	Reference	1.12 (0.78, 1.64)	0.98 (0.64, 1.50)	0.99 (0.83, 1.18)	**<0.001**
LM, kg	<35.00	35.00–38.60	≥38.70		
Case/total (%)	87/1071 (8.12)	105/1105 (9.50)	88/983 (8.95)		–
Model 1	Reference	1.19 (0.88, 1.60)	1.11 (0.82, 1.52)	**1.15 (1.02, 1.30)**	**<0.001**
Model 2	Reference	0.88 (0.61, 1.26)	0.70 (0.46, 1.07)	0.99 (0.82, 1.19)	**<0.001**

Data are presented as odds ratios with corresponding 95% confidence intervals. “Per 1 SD Increase” represents the effect value of GDM risk corresponding to each standard deviation increase in body composition; while “*P* for trend” is the trend effect value obtained by including body composition tertiles as ordinal variables in the regression model.

GDM, gestational diabetes mellitus; OR, odds ratio; CI, confidence intervals; SD, standard deviation; PBF, percentage of body fat; FM, body fat mass; FFM, fat free mass; LM, lean mass.

Model 1: without adjustment.

Model 2: adjusted for maternal age, early pregnancy body mass index, nationality, household registration type, education level, gravidity, parity, gestational weight gain. Bold values represent statistical significance.

**Figure 1:**
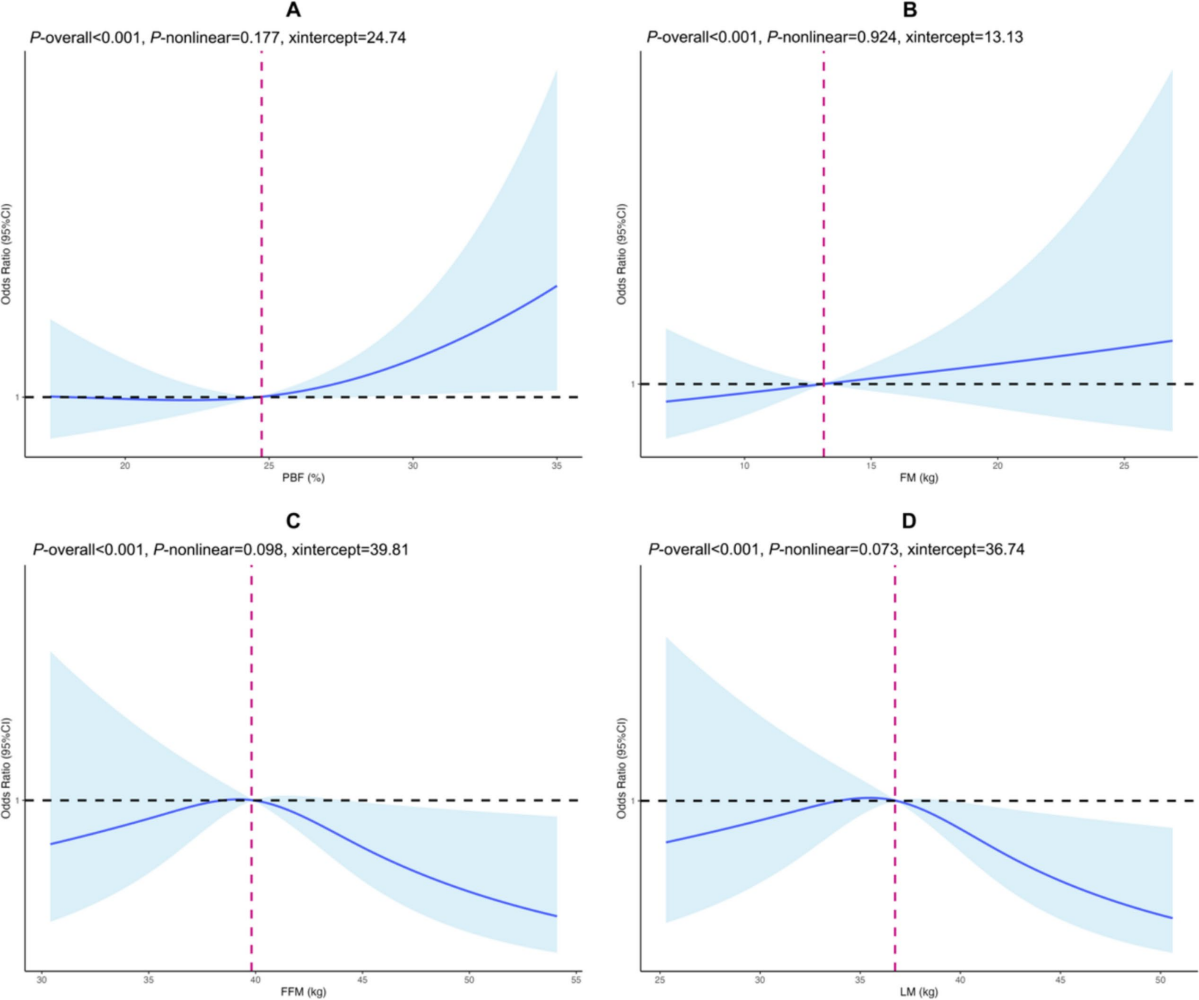
Cubic spline regression of body composition in early pregnancy and gestational diabetes mellitus. **(A)** PBF, **(B)** FM, **(C)** FFM, **(D)** LM. The horizontal axis represents the body component value (continuity variable), and the vertical axis is the odds ratio corresponding to GDM risk. The black horizontal dashed line is the reference line for the odds ratio, while the fuchsia vertical dashed line indicates the value of the body composition when OR is 1. Solid blue lines and shadows are point estimates with corresponding 95% confidence intervals of GDM risk. PBF, percentage of body fat; FM, body fat mass; FFM, fat free mass; LM, lean mass. Adjusted for early pregnancy BMI, maternal age, ethnicity, household registration type, education level, gravidity, parity, gestational weight gain.

The RCS analysis demonstrated that early-pregnancy PBF and FM levels were positively and linearly associated with GDM risk, with threshold values identified as 24.74% for PBF and 13.13 kg for FM. In contrast, early-pregnancy FFM and LM levels were negatively and linearly associated with GDM risk, with threshold values of 39.81 kg and 36.74 kg, respectively (*p*-overall < 0.001, *p*-non-linear range: 0.073–0.924) (Figure 1).

## Discussion

This large-scale cohort study (3,159 pregnant women) investigated the relationship between maternal body composition at 6–13 weeks of gestation and the risk of maternal GDM. Our findings revealed that elevated PBF and FM were independently associated with an increased risk of GDM, whereas elevated FFM and LM showed protective effects against the development of GDM.

In the literature, the association between obesity and GDM risk has mainly been assessed using traditional BMI (21, 22). In our study, the proportion of pregnant women with an early-pregnancy BMI ≥ 24 kg/m^2^ was higher in the GDM group than in the control group (29.29% vs. 19.66%), which aligns with previous findings (21, 22). Although BMI is a widely used parameter for assessing obesity, it cannot provide accurate information on fat distribution (23). In overweight individuals, BMI is highly correlated with the FM (24); however, some women with normal weight are also prone to abnormal body fat accumulation, a condition known as normal-weight obesity. A Korean study identified normal-weight obesity as an early predictive biomarker of metabolic syndrome (25). A prospective cohort study from China on the general population also showed that individuals with normal-weight obesity had a significantly increased risk of diabetes after 9 years (26). Moreover, evidence from the National Health and Nutrition Examination Survey indicates that within each BMI category, higher body fat levels are associated with increased homeostatic model assessment-insulin resistance (27). This suggests that even at a normal body weight, fat accumulation can induce changes in glucose metabolism, affecting health.

Positive associations between first-trimester PBF and FM levels and GDM risk were observed in our research. A retrospective study by Zhang et al. (28) similarly reported that elevated early-pregnancy PBF was an independent risk factor for GDM. Rahnemaei et al. (29) conducted a meta-analysis (29 studies, 56,438 participants) and found that FM, especially visceral fat, significantly contributes to GDM progression, consistent with our findings. Additionally, our study showed that for each standard deviation increase in first-trimester PBF and FM, the odds of developing GDM increased by 31 and 27%, respectively. This discovery fills a knowledge gap regarding dose–response relationships. Unlike previous studies, we verified the independence between BMI and body composition measurements, enhancing the reliability of risk assessment. This strengthens the validity of our findings and indicates that body composition parameters may provide greater value than traditional BMI in early pregnancy GDM risk assessment.

Restricted cubic spline analysis provided important thresholds for body composition parameters in predicting GDM risk. Skeletal muscle may protect against GDM by improving insulin sensitivity and regulating glucose metabolism (10, 30). The identification of specific thresholds (PBF: 24.74%, FM: 13.13 kg, FFM: 39.81 kg, LM: 36.74 kg) provides valuable clinical reference points. Notably, our determined PBF threshold (24.74%) was considerably lower than the typical lean obesity thresholds (30–35% body fat) for women cited in previous definitions (31). This lower threshold suggests that even a moderate elevation in early-pregnancy PBF may substantially impact GDM risk. A previous study (32) reported an average PBF of 33.18% ± 5.94% (our study: 25.16% ± 4.49%) among subjects aged 30.20 ± 3.98 years (our study: 27.77 ± 3.99 years). Age may influence PBF levels, and studying younger populations could help establish lower PBF thresholds.

A metabolic characteristic of GDM is relative insulin deficiency, wherein maternal β-cell insulin secretion cannot compensate adequately for the gradual increase in insulin resistance during pregnancy (33). Elevated circulating placental hormones, including estrogen, progesterone, and growth hormone, decrease insulin sensitivity, prompting β-cells to secrete more insulin and contributing to insulin resistance (34). Obesity before or early in pregnancy exacerbates insulin resistance. The complex mechanisms by which obesity induces metabolic disorders have been widely reported (35, 36). Adipose tissue is an active endocrine organ that can secrete various adipokines and cytokines, which induce chronic low-grade inflammation and insulin resistance, impairing glucose uptake in peripheral tissues (37, 38). High FM levels may further exacerbate insulin resistance (39, 40), leading to hyperglycemia and GDM.

Identifying PBF and FM as important predictors of GDM risk holds crucial clinical value. Early assessment of body composition helps identify women at a high risk of GDM before significant metabolic disorders occur. Early identification facilitates timely interventions, including personalized nutrition management (41), physical activity plans (42), and closer blood glucose monitoring (43), potentially reducing GDM risk or severity. We recommend incorporating body composition measurements into routine prenatal care to enhance GDM risk stratification and prevention.

This large-scale study assessed obesity’s impact on GDM using body composition rather than BMI, providing more accurate results than previous BMI-based studies. However, several limitations remain. First, using BMI measured at the first obstetric visit instead of pre-pregnancy BMI may not accurately reflect pre-pregnancy condition, potentially causing information bias. Second, confounding bias may still exist due to unmeasured or unknown variables. For instance, previous evidence suggests correlations between GDM incidence and dietary nutrition (fiber intake, vitamin D, sugar-sweetened beverages) (42, 44–46), physical activity (42), socioeconomic status (47), sleep duration (48), and mental health factors such as anxiety or depression (49). Additionally, we only collected body composition data in early pregnancy, thus, changes during pregnancy might have greater impacts on GDM risk. Finally, all participants were from southwestern China, limiting the generalizability of our findings to other populations. Further research is urgently needed to explore the mechanisms underlying the non-linear relationship between lean body mass and GDM risk.

## Conclusion

GDM risk is positively associated with PBF and FM but negatively correlated with FFM and LM. Body composition measurements should be incorporated into routine prenatal care to facilitate the early identification and prevention of GDM.

## Data Availability

The original contributions presented in the study are included in the article/Supplementary material, further inquiries can be directed to the corresponding author.
