# Supplementary material for: Association between body composition in early pregnancy and the risk of gestational diabetes mellitus
Source: Front Nutr. 2025 May 21;12:1565986. doi: 10.3389/fnut.2025.1565986 (PMC12133464; doi:10.3389/fnut.2025.1565986)
Supplement: Supplementary file 1 [file Table_1.docx]

## Supplementary Tables and figures

**Supplementary Table 1 Normality Analysis of Variables**

| Variables |  | **Kolmogorov-Smirnov test** | |
| --- | --- | --- | --- |
|  |  | W | *P values* |
| OGTT-fasting |  | 0.067 | <0.05 |
| OGTT-1 hour |  | 0.029 | <0.05 |
| OGTT-2 hour |  | 0.059 | <0.05 |
| PBF |  | 0.061 | <0.05 |
| FM |  | 0.082 | <0.05 |
| FFM |  | 0.051 | <0.05 |
| LM |  | 0.027 | <0.05 |

Abbreviations: OGTT, oral glucose tolerance test; PBF: percentage of body fat; FM: body fat mass; FFM: fat free mass; LM, lean mass.

**Supplementary Table 2** **Collinearity between body composition markers and body mass index**

| Body composition |  | Collinearity diagnostics | |
| --- | --- | --- | --- |
|  |  | VIF | Tolerance |
| PBF |  | 2.87 | 0.35 |
| FM |  | 4.68 | 0.21 |
| FFM |  | 1.96 | 0.51 |
| LM |  | 2.03 | 0.49 |

Abbreviations: PBF: percentage of body fat; FM: body fat mass; FFM: fat free mass; LM, lean mass; VIF: Variance Inflation Factor.

**
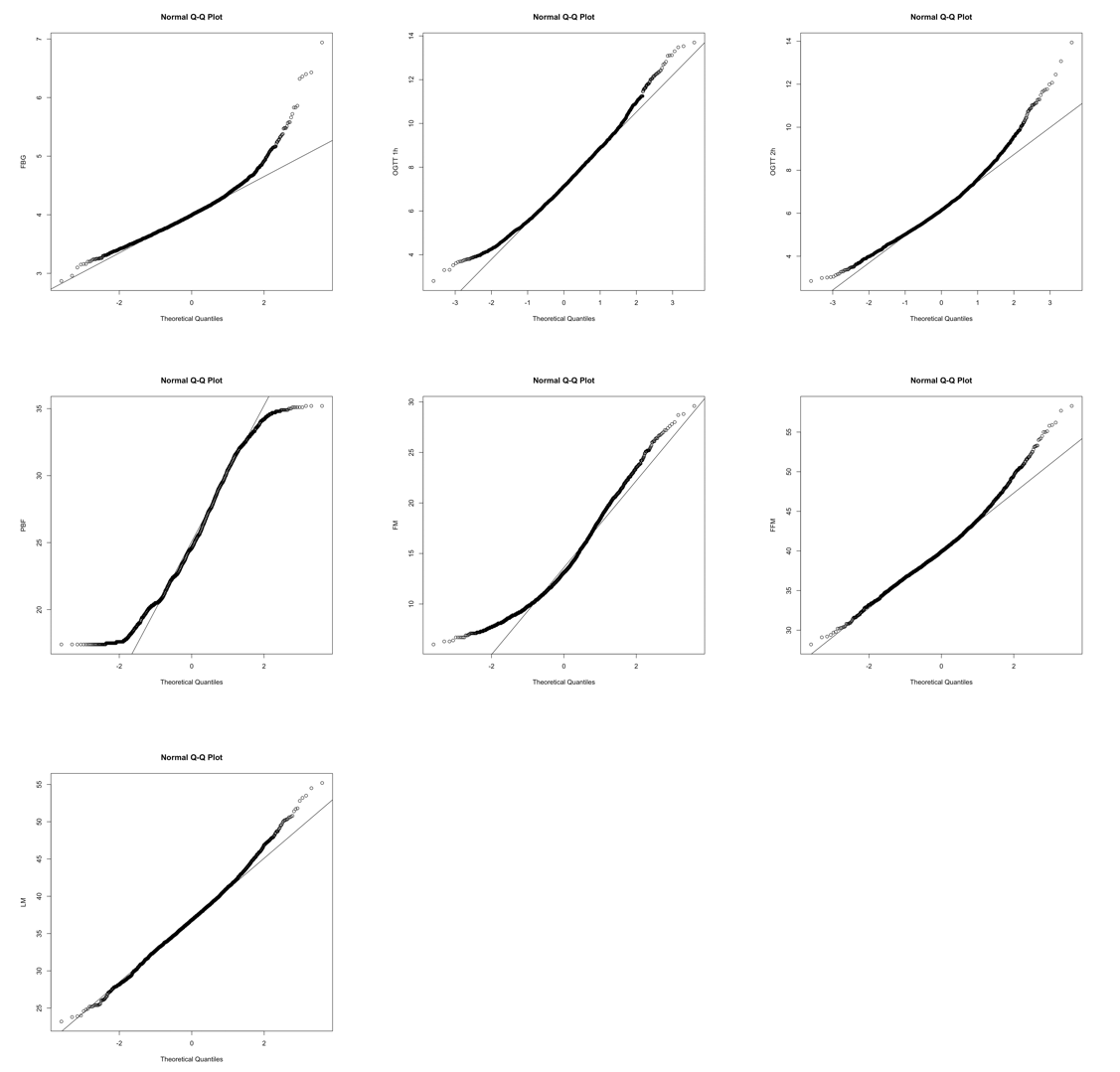
**

**Supplementary Figure 1 Normal Q-Q Plot** **of Variables**

Abbreviations: OGTT, oral glucose tolerance test; PBF: percentage of body fat; FM: body fat mass; FFM: fat free mass; LM, lean mass.
